# AI-driven discovery of blood xenobiotic biomarkers in neovascular age-related macular degeneration using iterative random forests

**DOI:** 10.1007/s00417-024-06538-2

**Published:** 2024-06-06

**Authors:** Steffen E. Künzel, Dominik P. Frentzel, Leonie T. M. Flesch, Vitus A. Knecht, Anne Rübsam, Felix Dreher, Moritz Schütte, Alexandre Dubrac, Bodo Lange, Marie-Laure Yaspo, Hans Lehrach, Antonia M. Joussen, Oliver Zeitz

**Affiliations:** 1 Department of Ophthalmology, Charité – Universitätsmedizin Berlin, corporate member of Freie Universität Berlin and Humboldt- Universität Zu Berlin, Campus Benjamin Franklin (CBF), Hindenburgdamm 30, 12203 Berlin, Germany; 2 Alacris Theranostics, Max-Planck-Straße 3, 12489 Berlin, Germany; 3 Département de Pathologie et Biologie Cellulaire, Université de Montréal, Montréal, QC H3C 3J7 Canada; 4 Max-Planck-Institute for Molecular Genetics, Ihnestrasse 63-73, 14195 Berlin, Germany

**Keywords:** Neovascular age-related macular degeneration, Peripheral blood xenobiotics, Modifiable risk factors, Intravitreal anti-VEGF injections, Treatment need, Machine learning, Personalized medicine, Systems biology, Iterative Random Forests, Artificial intelligence

## Abstract

**Purpose:**

To investigate the xenobiotic profiles of patients with neovascular age-related macular degeneration (nAMD) undergoing anti-vascular endothelial growth factor (anti-VEGF) intravitreal therapy (IVT) to identify biomarkers indicative of clinical phenotypes through advanced AI methodologies.

**Methods:**

In this cross-sectional observational study, we analyzed 156 peripheral blood xenobiotic features in a cohort of 46 nAMD patients stratified by choroidal neovascularization (CNV) control under anti-VEGF IVT. We employed Liquid Chromatography—Tandem Mass Spectrometry (LC–MS/MS) for measurement and leveraged an AI-driven iterative Random Forests (iRF) approach for robust pattern recognition and feature selection, aligning molecular profiles with clinical phenotypes.

**Results:**

AI-augmented iRF models effectively refined the metabolite spectrum by discarding non-predictive elements. Perfluorooctanesulfonate (PFOS) and Ethyl β-glucopyranoside were identified as significant biomarkers through this process, associated with various clinically relevant phenotypes. Unlike single metabolite classes, drug metabolites were distinctly correlated with subretinal fluid presence.

**Conclusions:**

This study underscores the enhanced capability of AI, particularly iRF, in dissecting complex metabolomic data to elucidate the xenobiotic landscape of nAMD and environmental impact on the disease. The preliminary biomarkers discovered offer promising directions for personalized treatment strategies, although further validation in broader cohorts is essential for clinical application.

**Supplementary Information:**

The online version contains supplementary material available at 10.1007/s00417-024-06538-2.


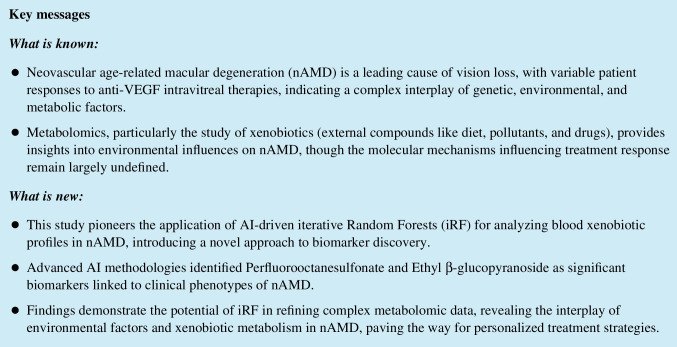


## Introduction

Age-related macular degeneration (AMD) stands as a principal cause of irreversible visual impairment and blindness among the elderly across the globe [1]. It is marked by the gradual degradation of the macula, the central portion of the retina, leading to a profound reduction in central vision [2]. While the management of AMD, particularly its neovascular form (nAMD) characterized by choroidal neovascularization (CNV), has been significantly advanced by regular anti-vascular endothelial growth factor (anti-VEGF) intravitreal treatment (IVT), patient responses to these treatments vary considerably [1, 2]. While some experience chronic activity, others attain effective CNV control after a small number of treatments, and maintain this retinal state under low-frequency anti-VEGF IVT [1–3].

The etiology of AMD is multifactorial, with major risk factors identified as advancing age, genetic predispositions, and environmental factors (like smoking or nutrition) [1, 2]. Despite a growing understanding of these risk factors, the precise molecular mechanisms influencing the variability in treatment response and disease progression remain elusive. Metabolomics offers a comprehensive approach to study the small molecules within biological systems and has emerged as a pivotal tool to unearth new biomarkers and gain deeper insight into disease mechanisms [4]. In the pursuit of understanding the environmental contributions to AMD, the study of xenobiotics – external compounds to which the body is exposed, such as dietary components, pollutants, and drugs – is particularly revealing [5]. These compounds can offer a window into the environmental influences that may exacerbate or mitigate the disease process.

Traditional univariate statistical methods, such as the Mann–Whitney U-test, have been the mainstay for identifying candidate molecules in disease association studies [6]. However, their ability to decode the complexities of multifaceted diseases like AMD is limited. Here, we propose the use of a multivariate machine learning technique, specifically iterative Random Forests (iRF), to navigate the intricate metabolic interplay. iRF can handle multiple variables in tandem, capturing the complex interactions between metabolites and clinical phenotypes that might otherwise be missed [7].

In short, this study utilizes iRF to analyze blood xenobiotic measurements from a stratified cohort of nAMD patients receiving anti-VEGF therapy, aiming to identify metabolites indicative of treatment response and to explore the influence of environmental factors on the disease. It further examines the clinical correlations of metabolites, grouped by compound class, to understand broad metabolic variations. This approach underscores the complexity of AMD’s metabolomic profile and the potential of machine learning in biomedical research, highlighting promising molecules and acknowledging the challenges in data analysis for future exploration.

## Materials and methods

### Study design

This investigation is a segment of a wider cross-sectional observational analysis aimed at identifying biomarkers for nAMD at the Charité University Hospital in Berlin, Germany. The execution of the research adhered strictly to the current versions of the study's protocol, Good Clinical Practice (ICH-GCP) Guidelines, and the principles set forth in the Declaration of Helsinki. Ethical clearance was granted by the appropriate ethics committee of the Charité University Hospital. All participants were required to give their written consent, having been prospectively enrolled for the study.

### Study protocol and subject recruitment

Between November 2018 and June 2020, at Charité University Hospital's Campus Benjamin Franklin, individuals who met the necessary criteria for inclusion without any of the exclusion factors were registered during their routine ophthalmology check-ups. A retrospective analysis of the medical records and imaging data was conducted for patients who had received anti-VEGF IVT within the six months prior to the study. To be included in the study, participants of either gender had to be over 51 years old, exhibit active subfoveal CNV due to nAMD in the eye under investigation, with a BCVA LogMAR between + 0.1 and + 1.3. In cases where both eyes qualified, the one with poorer visual acuity, or if equal, with clearer lens, less subfoveal scarring, or less geographic atrophy was chosen, following provision of informed consent. Exclusion criteria encompassed any form of CNV not associated with nAMD, subretinal hemorrhage necessitating surgical intervention besides anti-VEGF IVT, any counterindications for ongoing intravitreal therapy, or any potential conflicts of interest related to the study staff. During recruitment, participants were sorted into two stratification arms based on multiple functional and morphological parameters: The chronically active CNV (CAC) arm consists of 25 patients whose CNV did not reach a quiescent state under therapy. Specifically, this refers to signs of CNV activity in multimodal imaging under anti-VEGF IVT within intervals of 6 weeks or less. In contrast, there are 21 patients who exhibit a quiescent CNV status (effectively controlled CNV = ECC) during extended therapy intervals of 10 weeks or more.

### Clinical examination and *meta*-feature logging

The ETDRS protocol was utilized to assess the visual function in both the study and fellow eyes. Comprehensive ophthalmologic evaluations were conducted on all subjects, which included dilated fundus examinations. Imaging, encompassing Fundus Autofluorescence (FAF), Optical Coherence Tomography (OCT), Fluorescein Angiography (FA, all with Spectralis equipment), and OCT Angiography (ZEISS Angioplex), was performed by seasoned technicians adhering to standardized methods to maintain uniformity and superior image quality. For the annotation of meta-features including demographic (age, gender) and general health details (incl. medication regimens), data were meticulously collected from electronic health records and by anamnestic enquiry. The research team promptly entered all study data into the clinical software system.

### Sample collection and mass spectrometry analysis

Due to the detailed nature of this method, we included Supplemental Material 1.

### Statistical approach and data analysis

Due to the detailed nature of this method, we included Supplemental Material 1.

## Results

### Study population and clinical phenotypes

**Fig. 1:**
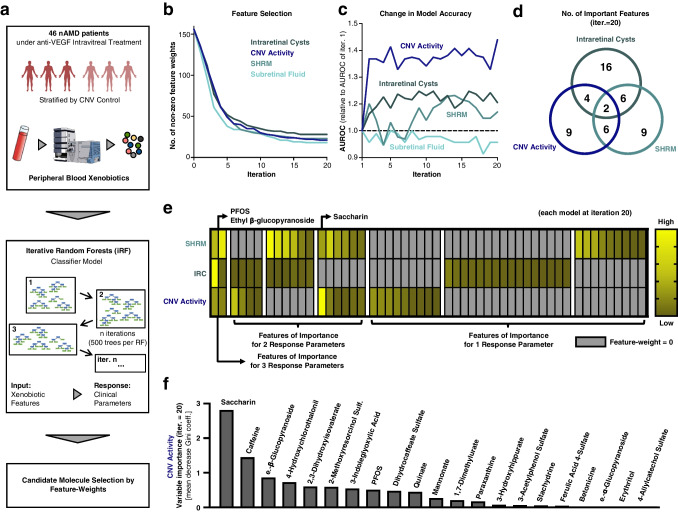
Iterative Random Forests uncovering interactions between peripheral blood xenobiotics and distinct clinical phenotypes in nAMD. **a**. Scientific Approach Overview: Peripheral blood samples were collected from a stratified group of 46 nAMD patients undergoing anti-VEGF IVT, and underwent LC–MS/MS mass spectrometry. Xenobiotic molecular features were selected for further analysis. iRF Classifier models, with 20 iterations and 500 trees each, utilized molecular features as inputs against defined clinical phenotypes as the output. Feature-weights (mean decrease of Gini coefficient) were used for molecular candidate selection. **b**. Feature-Weight Dynamics Across Iterations: The graph displays the count of non-zero feature-weights as a function of the number of iterations for four selected clinical phenotypes. A plateau in numbers is evident after 15–20 iterations, indicating stability in feature-weight counts. **c**. Model Performance Over Iterations: This component compares the model accuracy, measured by AUROC, across iterations relative to the baseline AUROC at the first iteration. The accuracy for identifying the presence of subretinal fluid shows no significant enhancement with additional iterations. **d.** Intersection of Influential Molecules: a Venn diagram presents the number of molecules with non-zero feature weights in the classification model after 20 iterations, with color-coding corresponding to the clinical phenotypes detailed in parts b and c. **e**. Phenotype-Specific Feature Weights Visualization: This segment graphically represents feature weights for the three phenotypes where iterative modeling marked a considerable improvement. Each phenotype is normalized to the highest value per model, such as Saccharin for CNV activity. Key molecules are annotated within the figure. **f.** Comprehensive Feature Analysis for Best-Performing Model: The figure illustrates all non-zero feature weights for the top-performing model concerning CNV activity. It quantifies variable importance using the mean decrease in the Gini coefficient, with all molecules labeled for reference

In our study, we stratified 46 nAMD patients into two cohorts based on the level of choroidal neovascularization (CNV) activity while they were receiving anti-VEGF intravitreal therapy (IVT, Methods, Fig. 1a). As previously reported, this stratification resulted in two groups: one characterized by chronically active CNV (CAC, 25 patients) and the other by effectively controlled CNV (ECC, 21 patients) [8, 9]. In addition to blood sampling, patients underwent thorough clinical and anamnestic examinations. As expected and also recently reported, the CAC and ECC patient groups significantly differed in multiple clinical features, e.g. in central retinal thickness (CRT, CAC: 328.9 µm ± 74.3 µm vs. ECC: 274.8 µm ± 45.7 µm, mean ± standard deviation, *p* = 0.0025), the frequency of anti-VEGF IVT (CAC: 4.32 weeks, w. ± 0.61 w. vs. 12.24 w. ± 3.1 w.; stratification criterion; *p* < 0.0001), and presence of subretinal hyperreflective material (SHRM, CAC: presence in 21/25 = 84% vs ECC: 5/21 = 23.8%, *p* < 0.0001). Presence of subretinal fluid (SRF, CAC: 17/25 = 68% vs ECC: 12/21 = 57.1%, *p* = 0.056) and intraretinal cysts (IRC; CAC: 13/25 = 52% vs ECC: 9/21 = 42.9%, *p* = 0.29) showed a tendency, albeit not being statistically significant. Furthermore, and as expected, presence of SHRM and IRC (18 patients positive for both, 14 negative for both, and only 5 patients with IRC but without SHRM, and 9 with SHRM but no IRC, *p* = 0.004) were not statistically independent.

### Iterative random forest classifier as a multivariate alternative approach for candidate molecule detection

The patients' blood was subjected to LC–MS/MS mass spectrometry for metabolomic analysis, which resulted in the detection of 899 distinct metabolites. From these, we selected 156 molecules identified as xenobiotics. For statistical analysis, we utilized the multivariate iterative Random Forests (iRF) approach with the molecular data as input and selected clinical phenotypes (CNV activity, SHRM, SRF, and IRC, one model for each of these phenotype dimensions) as binary response variables (see Methods section for details). In brief: We employed iRF similarly as previously described [7], with 500 trees per Random Forest (RF) and 20 iterations, and a 50:50 data split for training and testing (Fig. 1a). The importance of individual metabolites (weights) for decision-making was measured by the mean decrease in the Gini coefficient. This parameter starts with equal importance for all metabolites (before iteration 1) and is then iteratively adjusted. It is noteworthy that after just a few iterations, many metabolites drop to zero weights across all four models. Furthermore, the number of non-zero feature weights for all models stabilizes at a constant after approximately 15–20 iterations: 21 metabolite features for IVT frequency, 28 for IRC, 22 for SHRM, and 19 for SRF (Fig. 1b, d). Additionally, the accuracy of the models (measured by the Area Under the Receiver Operating Characteristic, AUROC of the classifiers) remains at a level that is not very stable (due to the small dataset) but does not improve further (Fig. 1c). Based on these results from Fig. 1b and c, we opted for 20 iterations but trained the algorithm for up to 200 iterations to exclude possible improvements with more iterations (not shown). Moreover, the classification model for SRF never showed improvement through the exclusion of individual molecular features, leading us to exclude the SRF model from further analyses in this iRF setting.

An overview of all non-null features for all three relevant models is shown as Suppl. Table 2. As we aim to capture various dimensions of the disease, we decided to focus particularly on candidate molecules that are crucial for different dimensions of the condition. In total, we identified 2 molecules that are decisive for all three dimensions (CNV activity, IRC, and SHRM): Perfluorooctanesulfonate (PFOS) and Ethyl β-glucopyranoside (Fig. 1d, e). Four molecules are important for the classifier for IRC and CNV activity, six are important for SHRM and CNV activity, and six for IRC and SHRM (Fig. 1e). The best classifier model is for CNV activity (Fig. 1c). For this, we have displayed all crucial molecules in order of importance (Fig. 1f).

### Stratification of metabolites by compound class and their clinical correlations

**Fig. 2:**
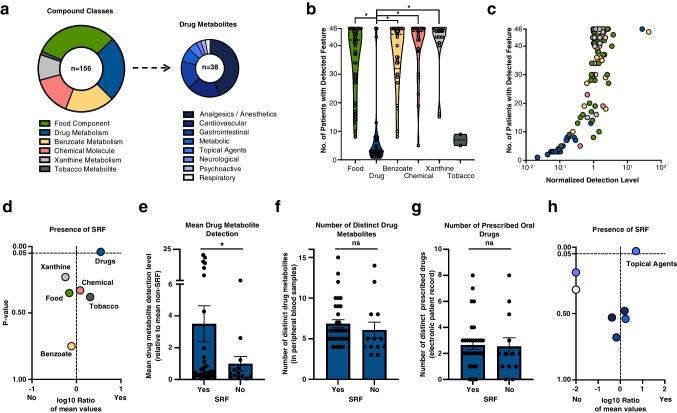
Grouped Analysis of Xenobiotic Molecules Elucidates Their Association with Subretinal Fluid in Neovascular AMD **a**. Categorization of Xenobiotic Molecules: We classified 156 xenobiotic molecules into six distinct compound classes. Of these, 38 molecules were related to drug metabolism and were further subdivided into eight specific drug families. The color-coding in part (a) corresponds to the entire figure. **b**. Detection Spread Across Compound Classes: This section illustrates the variance in detection frequencies of the six compound classes across the cohort. Statistical significance was assessed between the ‘Drug’ class and all other classes, except 'Tobacco' due to its small sample size (only two samples). **c**. Normalized Detection Levels and Patient Counts: The graph plots the normalized levels of detected metabolites against the number of patients in whom each feature was detected. **d**. Volcano Plot for Compound Class Association with subretinal fluid (SRF) presence: A volcano plot showcases whether the mean detection levels of xenobiotic compound classes feature on SRF detection. **e.-g**. Comparative Analysis between patients with and without presence of SRF: Mean detection levels of drug metabolites (e), count of distinct drug metabolites (f), number of prescribed oral drugs (g) were compared between the two patient groups. **h**. Volcano Plot for Drug Subclass Association with SRF Presence: A second volcano plot, similar in design to (d), evaluates the association of drug sub-classes on the presence of SRF in patients. For parts (d) to (h), the Mann–Whitney U-test was applied to determine statistical significance, with a p-value threshold of less than 0.05. The analysis also included the Benjamini–Hochberg procedure for FDR correction

We also categorized the 156 molecules based on their structure and occurrence into six compound classes. As anticipated, the majority of molecules were attributable to food metabolism (49 distinct molecules, 31.4%). Also expected in an elderly population was a frequent presence of molecules associated with drug metabolism (38, 24.4%). Following these were molecules linked to benzoate metabolism (29, 18.6%), chemical molecules (23, 14.7%), xanthine metabolism (15, 9.6%), and tobacco use (2, 1.3%). Molecules associated with drugs can be further divided into eight subgroups, with analgesics/anesthetics being the most common (17/38, 44.7%, Fig. 2a).

Upon closer examination of how frequently each molecule occurs among patients, it is noticeable, with few exceptions, that metabolites of drugs are found in the blood of only a few rather than many patients—this is in significant contrast to all other compound classes, except for tobacco, which was also found in fewer than 10 patients (Fig. 2b). Similarly, normalized measurement values for drug-associated metabolites are significantly lower compared to those of other compound classes (Fig. 2c).

We then addressed the question of whether individual compound classes are generally associated with certain phenotypes. The only statistically significant correlation was found for drug-associated metabolites, which, on average, were statistically higher in patients with SRF (Fig. 2d, e). Interestingly, this correlation pertains only to the level of measurement values (Fig. 2d, e), not to the number of different drug-associated metabolites (Fig. 2f) or the number of prescribed oral drugs (Fig. 2g). A further breakdown of these metabolites reveals that topical agents are associated with increased SRF in AMD. For anti-VEGF, SHRM, and IRC, we were unable to demonstrate any significant correlations in this analysis (not shown).

## Discussion

To the best of our knowledge, this is the first study to explore xenobiotics in the context of AMD. We report three core findings: 1. We demonstrate that AI driven, multivariate methods like iRF can be valuable in analyzing especially data-sparse datasets. 2. We identify new molecules that may be associated with the disease. 3. We show that a general predominance of a compound metabolite class is not associated with a generally better or worse phenotype (except for drug metabolites and SRF).

We utilize the approach of iRF, which confers several notable advantages in our context. iRFs adeptly handle multivariate data and mitigate the risk of overfitting, a critical consideration in datasets with high feature-to-sample ratios. They excel in feature selection even within sparse data landscapes, through an iterative process that progressively refines the significance of each variable according to its contribution to model accuracy. Thereby, iRFs construct numerous decision trees during training and output the mode of the classes for classification of the individual trees. Diverging from standard random forests, iRFs apply iterations to progressively concentrate on the most informative features, eliminate those that add noise or are redundant and prevent from wrong conclusions from outliers [7]. In our scenario, this iterative feature refinement enables a focus on metabolites that exhibit consistent importance across iterations, thereby identifying potential biomarkers or therapeutic targets with higher fidelity. Adding to this, such an iterative approach aligns more closely with biological understanding, particularly in the context of angiogenesis in AMD, where numerous molecules interact [1, 2]. iRFs can detect these complex interactions, unlike univariate canonical analyses, which often overlook the interplay between multiple factors. This makes iRFs not only statistically robust but also biologically more intuitive, as they consider the multifactorial nature of biological processes like the growth of blood vessels, thereby offering insights that are both scientifically relevant and biologically plausible.

The most significant result highlights two molecules of importance across all three models: PFOS and Ethyl β-glucopyranoside (Fig. 1d, e). PFOS and similar metabolites have already been associated with the occurrence of retinal diseases and specifically with AMD in the past [10–13]. Studies indicate that PFOS not only induces oxidative stress but also triggers inflammatory reactions — both processes significantly involved in AMD [1]. PFOS was primarily used to impregnate materials such as textiles, carpets, and paper to make them resistant to grease, oil, and water. Additionally, it has been used in chrome plating, analog photography, older fire-fighting foams, and hydraulic fluids for the aerospace industry. Despite being banned and added as a pollutant to Annex B of the Stockholm Convention in 2009, PFOS continues to be emitted into the environment, mainly from metal processing (chrome plating) and fire-fighting foams [14, 15]. It is surprising and concerning that we have identified PFOS as significant for various phenotypes.

Ethyl β-glucopyranoside, as the name implies, belongs to the group of glucosides, or more broadly, glycosides. This is highly intriguing, as glycosides also have strong associations with AMD and retinal health as shown in numerous studies [16–21], with cardiac glycosides (e.g. Digoxin) being a prominent example known for retinotoxicity [22]. On the contrary, glucosides could also have a protective character in retinal disease, as suggested by other studies [20]. Potential mechanisms include: attenuation of oxidative stress, disruption of cellular signaling pathways essential for retinal health, leading to cellular dysfunction and degeneration, as well as interference with the normal metabolic processes in the retina, contributing to an accumulation of toxic byproducts that exacerbate retinal damage [23–25]. The specific implications for these two molecules remain unclear, but we consider the finding of them confirmatory for our model, together with the identification of additional molecules that have also already been associated with certain AMD and retina phenotypes (incl. Saccharin [26–28], Quinate [29, 30], Paraxanthine [31], 3-Acetylphenol Sulfate [21], Ferulic Acid 4-Sulfate [32], Ethyl α-glucopyranoside, and Erythritol [26–28], Fig. 1f). Moreover, it is important not to be misled by smaller weights in our iRF classifier, as they can tip the scales in determining the ultimate classifier outcome (Fig. 1f).

In our second analytical approach (Fig. 2), we categorized the individual xenobiotic molecules into various compound classes and examined their correlation with clinical phenotypes. An initial interesting finding is that only two molecules associated with tobacco consumption appear here—while smoking is generally understood as an independent risk factor for AMD [1] (Fig. 2a-b). Additionally, the heterogeneous occurrence of drug-associated molecules (both in number and generally lower measurement values) is noteworthy (Fig. 2b-c). We consider such investigations to be very important, especially in an old and aging cohort with polymorbidity and polypharmacy like in AMD patients. Interpreting the result where higher measurement values of drug-associated metabolites are found in patients with SRF, but not a greater variety of such metabolites, and also considering that these patients do not report a higher number of prescribed oral medications, is currently challenging for us (Fig. 2e-g). This is particularly perplexing given that the role of SRF in nAMD has not yet been conclusively established [1, 2, 33]. We speculate that the elevated levels of certain drug-associated metabolites (e.g. topical agent metabolites) in patients with SRF could be indicative of a localized tissue response to therapy or a specific metabolic pathway alteration associated with the disease's progression. However, this remains a research avenue that requires further exploration.

Nevertheless, it should be noted that the data presented here are preliminary. We caution against drawing clinical conclusions or changing lifestyle behaviors based on these early findings due to several limitations: our dataset has very low data density with a small sample size compared to a larger number of metabolomic dimensions. Given this context, it is questionable whether our statistical approach is exceptionally robust in this setting, which is why we are pursuing external validation (discussed below). Furthermore, iRF, like many machine learning models, are 'black boxes' which means we do not fully understand how the molecules influence the classifier’s decisions. What we also do not know is how well the algorithm performs compared to other algorithms, especially in a real-world setting. At this point, the study lacks external validation or molecular biological investigation in animal models – which we think will be critical for clinical use in the future. As an additional limitation, particularly regarding reproducibility, it must be mentioned that although we made every effort to ensure consistent measurements (such as blood collection at the same time of day and employing robust statistical methods), we cannot entirely prevent variation between participants and timings. Given that the metabolites in question have been scarcely or not at all studied in humans, we cannot find data on their half-lives or similar parameters. This makes it difficult for us to confidently state whether the results can be reliably reproduced. Other limitations include the potential selection bias, and unanalyzed confounding factors, which could misrepresent the true effects of the molecules, and the absence of longitudinal analysis further constrains the implications of our study.

In summary, our study leverages the power of AI through multivariate iRF models to uncover key xenobiotic molecules potentially associated with nAMD. The application of iRF models has allowed us to navigate a complex multidimensional dataset to a degree unattainable with conventional statistical methods. This advanced AI analysis has illuminated molecular candidates that are potentially associated with AMD. Investigation of compound classes has shown that no single class exerts a solely positive or negative impact, although drug metabolites appear to influence the presence of SRF. These insights, while still in the preliminary stages, significantly enhance our understanding of the intricate metabolomic interactions present in AMD and set a foundation for future studies to validate and further clarify these associations.

## Supplementary Information

Below is the link to the electronic supplementary material.

Supplementary file1 (DOCX 35 KB)

Supplementary file2 (XLSX 85 KB)

Supplementary file3 (XLSX 12 KB)

## Data Availability

Raw data can be found as Suppl. Table 1.

## Abbreviations and Acronyms

AUROC: Area Under the Receiver Operating Characteristics
anti-VEGF: Anti-vascular endothelial growth factor
BCVA: Best-corrected visual acuity
CAC: Chronically active CNV
CNV: Choroidal neovascularization
Coeff.: Coefficient
ECC: Effectively controlled CNV
ETDRS: The Early Treatment Diabetic Retinopathy Study Group
FAF: Fundus autofluorescence
FA: Fluorescein angiography
FDR: False Discovery Rate
IRC: Intraretinal cysts
iRF: Iterative Random Forests
IVT: Intravitreal treatment
LC–MS/MS: Liquid chromatography with tandem mass spectrometry
nAMD: Neovascular age-related macular degeneration
OCT: Optical coherence tomography
SHRM: Subretinal hyperreflective material
SRF: Subretinal fluid
sulf.: Sulfate

## Code Availability

Analysis codes will be shared by the corresponding author on reasonable request.
